# Clinical and Haematologic Correlates of Soluble VCAM‐1 in Adults With Steady‐State Sickle Cell Disease

**DOI:** 10.1002/jha2.70413

**Published:** 2026-10-22

**Authors:** William Kwesi Ghunney, Eugenia Vicky Asare, John Benaiah Ayete‐Nyampong, Wonder Doku‐Agama, Eric Kyei‐Baafour, Michael Fokou Ofori, George Obeng Adjei, Edeghonghon Olayemi

**Affiliations:** ^1^ Ghana Institute of Clinical Genetics Accra Ghana; ^2^ Department of Haematology Korle‐Bu Teaching Hospital Accra Ghana; ^3^ College of Health Sciences University of Ghana Accra Ghana; ^4^ Centre for Tropical Clinical Pharmacology and Therapeutics University of Ghana Medical School Accra Ghana; ^5^ Department of Immunology Noguchi Memorial Institute for Medical Research Accra Ghana; ^6^ Department of Haematology University of Ghana Medical School Accra Ghana; ^7^ Vancouver Coastal Health Richmond Canada

**Keywords:** body mass index, endothelial activation, reticulocytosis, sickle cell disease, steady state, systolic blood pressure, vascular cell adhesion molecule‐1

## Abstract

**Background:**

Endothelial activation contributes to the vasculopathy of sickle cell disease (SCD). We evaluated serum soluble vascular cell adhesion molecule‐1 (sVCAM‐1), a surrogate marker of endothelial activation, and examined its clinical and haematologic correlates in adults with steady‐state SCD.

**Methods:**

This was a cross‐sectional study of 75 adults with steady‐state SCD (HbSS: 86.7%; HbSC: 13.3%) and 75 age‐matched comparison participants with normal adult haemoglobin genotype (HbAA). Associations of clinical and haematologic measures with sVCAM‐1 were evaluated using correlation and multivariable linear regression analyses.

**Results:**

SCD participants had lower systolic blood pressure (SBP), BMI and haemoglobin and elevated leukocyte, platelet, reticulocyte, total bilirubin and LDH levels, compared with HbAA participants (all *p *< 0.001). Mean sVCAM‐1 was higher in SCD than in HbAA participants (878.8 vs. 507.9 ng/mL; *p *< 0.001), with no significant difference between HbSS and HbSC. In the combined SCD cohort, sVCAM‐1 correlated positively with absolute reticulocyte count (*r* = 0.24, *p = *0.034). In HbSS, sVCAM‐1 correlated positively with SBP (*r* = 0.264, *p = *0.033). In multivariable analysis, higher reticulocyte count (B = 73.39 ng/mL per 100x10^9^/L increase, *p = *0.014) and lower BMI (B = −15.3, *p = *0.038) were independently associated with higher sVCAM‐1 in SCD. In HbSS, total bilirubin was inversely associated with sVCAM‐1 in the primary model (B = −1.62, *p = *0.041), although this association was attenuated in sensitivity analyses.

**Conclusion:**

Higher reticulocyte count was consistently associated with higher sVCAM‐1 in adults with steady‐state SCD. Associations of sVCAM‐1 with BMI, bilirubin and SBP were also observed, but these genotype‐specific findings warrant cautious interpretation, particularly given the small size of the HbSC subgroup. These findings support further prospective evaluation of clinical and haematologic correlates of endothelial activation in SCD.

**Trial Registration:**

The authors have confirmed clinical trial registration is not needed for this submission

## 1 Introduction

Sickle cell disease (SCD) is an inherited disorder controlled by a single pair of genes; however, it is also influenced by polygenic and environmental factors [1]. It is one of the most prevalent inherited haemoglobinopathies worldwide, with the highest disease burden in sub‐Saharan Africa, where up to 300,000 infants are born annually with the condition [2, 3]. In Ghana, approximately 2% of newborns are delivered each year with SCD [4]. Vascular complications are central to its clinical expression, driven in part by chronic haemolysis, inflammation and endothelial activation [5].

In SCD, normal haemoglobin (HbA) is replaced by sickle haemoglobin (HbS) along with another ‘variant’ haemoglobin [6]. HbS polymerizes when deoxygenated, damaging the erythrocyte and resulting in the loss of cations and water. The sickled erythrocytes exhibit abnormal rheological features, and the tendency towards vaso‐occlusion characteristically leads to ischemic damage, resulting in acute pain or organ damage [7]. In plasma, free Hb disrupts NO and generates free radicals [8]. During haemolysis, this depletion of NO is further aggravated by the release of arginase from erythrocytes into plasma, which destroys L‐arginine, which is used by endothelial NO synthase as a substrate in its reactions [9, 10]. Pathological stimulation of endothelial cells results in the expression of adhesion molecules such as vascular cell adhesion molecule‐1 (VCAM‐1 [CD106]), an inducible 90‐kDa glycoprotein. This occurs when NO bioavailability is impaired, leading to endothelial dysfunction [11, 12]. The adhesion molecules on the surface of these cells, which are shed into the plasma as soluble molecules, serve as useful markers of endothelial dysfunction, inflammation and endothelial activation [11]. Endothelial activation in SCD extends beyond VCAM‐1 expression and involves leukocyte and platelet adhesion, selectin signalling, activation of coagulation pathways, dysregulation of von Willebrand factor, endothelial injury and the generation of circulating microparticles derived from erythrocytes, platelets and endothelial cells [13, 14, 15]. These processes promote inflammation, thrombin generation and vascular dysfunction, while abnormalities in natural anticoagulant pathways, including protein C and protein S, may further contribute to the prothrombotic state [14, 15, 16]. Although these pathways were not directly evaluated in the present study, they provide the broader pathophysiologic context in which sVCAM‐1 should be interpreted. Serum sVCAM‐1 has been shown to be significantly elevated in HbSS individuals in steady state compared with the non‐SCD comparison group. Increased steady‐state levels have been associated with an increased risk of end‐organ damage and premature mortality in these individuals [11, 17]. However, the determinants of VCAM‐1 expression in African populations living with SCD remain incompletely defined.

Several biological processes have been implicated in modulating endothelial activation in SCD. Reticulocytosis, reflecting increased erythropoietic drive and haemolysis, contributes to vascular injury by exposing adhesive surface molecules [18, 19, 20, 21]. Markers of haemolysis, such as lactate dehydrogenase (LDH) and bilirubin, have also been linked to endothelial stress; however, their associations with adhesion molecules are inconsistent [11, 22, 23]. Nutritional status, often compromised in SCD and reflected by lower body mass index (BMI), may further exacerbate vascular dysfunction [24, 25]. In addition, vascular factors, such as systolic blood pressure (SBP), have been shown to influence endothelial biology; however, their relationship with adhesion molecule expression in SCD has not been fully explored [26, 27].

Although elevated circulating sVCAM‐1 has previously been reported in individuals with SCD, including in Ghanaian cohorts [17], its clinical and haematologic correlates in adults with steady‐state SCD remain incompletely characterized in sub‐Saharan Africa. In particular, the relationships of sVCAM‐1 with reticulocytosis, haemolytic markers, nutritional status and blood pressure require further study. We therefore evaluated clinical and haematologic correlates of sVCAM‐1 in adults with steady‐state SCD.

## 2 Methods

### 2.1 Study Design

This was a cross‐sectional study conducted among adults with SCD at the Ghana Institute of Clinical Genetics (GICG). This study was approved by the Scientific and Technical Committee and the Institutional Review Board of the Korle‐Bu Teaching Hospital (KBTH‐STC/IRB/000217/2021). In addition, institutional approvals were obtained from the GICG and the Southern Area Blood Centre (SABC) of the National Blood Service of Ghana.

### 2.2 Study Sites

The study sites were the GICG, located on the premises of the Korle‐Bu Teaching Hospital, and the Donor Clinic of the SABC of the National Blood Service, Ghana (NBSG). The GICG has over 25,000 registered patients with SCD. Every year, the clinic records between 10,000 and 15,000 clinic visits, with an average daily attendance of almost 50 patients [28].

### 2.3 Study Population

The study enrolled 75 individuals with SCD who had accessed outpatient services at the GICG for at least 1 year prior to recruitment and provided informed consent, as well as a comparison group of 75 age‐matched HbAA voluntary blood donors who were accessing services at the Donor Clinic of the SABC of the NBSG.

#### 2.3.1 Inclusion Criteria

Eligible participants included individuals with a confirmed Hb genotype (HbSS, HbSC and HbAA) who were aged 18 years or older. Individuals with SCD were eligible if they were in steady‐state according to the criteria described by Ballas et al. [29]. Operationally, participants should not have had an acute vaso‐occlusive event requiring emergency or hospital treatment for at least 4 consecutive weeks; no intercurrent infection or inflammatory illness during the previous 4 weeks; no recent hospitalization; and no blood transfusion during the 4 months preceding enrolment. Participants were also included if they had not received medications that could alter haematologic parameters, such as antibiotics, during the previous 3 weeks.

#### 2.3.2 Exclusion Criteria

SCD patients with Hb genotypes other than HbSS and HbSC were excluded. Blood donors with Hb genotypes other than HbAA were also excluded. SCD patients who were on chronic blood transfusion therapy, or enrolled in a clinical trial and received an investigational medicinal product during the study period, as well as those who had participated in a clinical trial and received an investigational medicinal product within the previous 6 months, were excluded.

### 2.4 Study Procedures

Study participants were recruited using the systematic random sampling method at both sites after obtaining informed consent. Structured questionnaires were used to interview all consenting participants, and the following variables were recorded:

- ***Demographic data***: age, sex
- ***Haemoglobin genotype***: SS, SC or AA
- ***Clinical measures***: brachial systolic and diastolic blood pressures, pulse rate, peripheral haemoglobin oxygen saturation, respiratory rate, weight and height.
- ***Complications of SCD***: acute vaso‐occlusive pain requiring emergency room attendance in the previous 12 months, history of acute chest syndrome, nephropathy, retinopathy, anaemia requiring transfusion, avascular necrosis, pulmonary hypertension, priapism, cardiomyopathy, sepsis/infection, gallstones, stroke, hospitalizations and chronic leg ulcers.
- ***Medical History***: hypertension, diabetes and medications (hydroxyurea, antihypertensives and antidiabetics).

### 2.5 Laboratory Analysis

The Sysmex XN‐450 Haematology Analyzer (Sysmex Corporation, Japan) was used to perform the full blood count (FBC) and reticulocyte counts. Hb electrophoresis was done for all participants using an alkaline medium (pH 8.2–8.6). The HumaStar 100 Clinical Chemistry Analyzer (Human Diagnostics, Germany) was used to measure serum LDH, total bilirubin and serum creatinine. The Human VCAM‐1/CD106 DuoSet ELISA Kit (Fisher Scientific, USA) was used to determine sVCAM‐1 levels. Concentrations were corrected for the sample dilution factor and are reported in ng/mL.

### 2.6 Statistical Analysis

Data were analysed using SPSS software version 23. Continuous variables were summarized as means (standard deviations [SD]) when normally distributed and as medians (interquartile range [IQR]) when non‐normally distributed. Categorical variables were summarized as frequencies and percentages. Between‐group comparisons of continuous variables were performed using the independent‐samples *t*‐test or Mann–Whitney *U* test, as appropriate, while categorical variables were compared using the chi‐square test or Fisher's exact test.

Associations between sVCAM‐1 and demographic, clinical and laboratory variables were evaluated primarily using Pearson's correlation coefficients. Analyses were performed in the combined SCD cohort and were additionally stratified by genotype (HbSS and HbSC); corresponding analyses were performed in the HbAA comparison group where appropriate. Because the HbSC subgroup included only 10 participants, all HbSC‐specific analyses were considered exploratory and interpreted cautiously. Associations between systolic blood pressure (SBP) and selected markers of haemolysis were assessed in a similar manner. Associations between individual SCD complications and sVCAM‐1 concentrations were evaluated using point bi‐serial Pearson correlation. The Kruskal‐Wallis test was used to compare sVCAM‐1 concentrations across vaso‐occlusive pain frequency categories.

To identify factors independently associated with sVCAM‐1, reduced multiple linear regression models were fitted with sVCAM‐1 concentration as the dependent variable. The primary model in the combined SCD cohort included sex, systolic blood pressure, total bilirubin, absolute reticulocyte count and BMI; the same model was evaluated in the HbSS subgroup. Absolute reticulocyte count was reported in ×10^9^/L and rescaled to represent increments of 100×10^9^/L in the regression models. The number of covariates was restricted because of the modest sample size and to reduce the risk of model overfitting. Multicollinearity was assessed using variance inflation factors (VIFs). Model fit was summarized using *R* ^2^, adjusted *R* ^2^, the overall F‐test, Akaike information criterion (AIC) and Bayesian information criterion (BIC).

Sensitivity analyses were performed to assess the robustness of the principal findings. Spearman's rank correlations were used to evaluate associations without assuming linear relationships or normally distributed variables. Because the distribution of sVCAM‐1 showed asymmetry, a Box–Cox transformation was additionally applied, and Pearson's correlations were repeated using the transformed outcome. Alternative multivariable regression specifications were also examined, including a model that replaced sex with age and a more parsimonious model that excludes both age and sex. Results of these sensitivity analyses are presented in the Supporting Information. All statistical tests were two‐sided, and *p* < 0.05 was considered statistically significant.

## 3 Results

### 3.1 Socio‐Demographic Characteristics

The median ages were 32 years (IQR 14) in the SCD cohort and 33 years (IQR 11) in the HbAA cohort (*p* =  0.098). Among SCD participants, 26/75 (34.7%) were male, and 49/75 (65.3%) were female (Table 1). However, in the HbAA comparison group, 63/75 (84%) were males, and 12/75 (16%) were females, consistent with the predominantly male voluntary blood donor population in Ghana. Of the SCD participants, 65/75 (86.7%) had HbSS, while 10/75 (13.3%) had HbSC (Table 1). None of the participants were on hydroxyurea or on chronic blood transfusion therapy. None of the participants were on antihypertensive or antidiabetic medication.

**TABLE 1:** Demographic characteristics and clinical measures for SCD participants and HbAA comparison group.

Variables	Cases, (*N* = 75)	Comparison group, (*N* = 75)	*p*‐value
Age, *n* (%) (years)			
18–40	64 (85.3%)	58 (77.3%)	
> 40	11 (14.7%)	17 (22.7%)	
Median age (IQR)	32 (14)	33 (11)	0.098
Sex, *n* (%)			**< 0.001**
Male	26 (34.7%)	63 (84.0%)	
Female	49 (65.3%)	12 (16.0%)	
Genotype (%)			
HbSS	65 (86.7%)		
HbSC	10 (13.3%)		
Mean weight (SD) (kg)	58.6 (11.0)	75.5 (15.5)	**< 0.001**
Mean height (SD) (m)	1.6 (0.1)	1.7 (0.1)	0.173
Mean BMI (SD) (kg/m^2^)	21.4 (3.6)	26.6 (6.6)	**< 0.001**
Median SBP (IQR) (mmHg)	112 (17)	132 (16)	**< 0.001**
Median DBP (IQR) (mmHg)	62 (11)	82 (15)	**< 0.001**
MAP (SD) (mmHg)	79.8 (8.5)	97.4 (7.2)	**< 0.001**

*Note*: Data are mean (SD), median (IQR), or *n* (%). Between‐group comparisons used *t*‐test or Mann–Whitney *U* test as appropriate; categorical variables used *χ* ^2^ or Fisher's exact test (two‐sided). Bold *p*‐values indicate *p* < 0.05.

Abbreviations: BMI, body mass index; DBP, diastolic blood pressure; MAP, mean arterial pressure; SBP, systolic blood pressure.

### 3.2 Clinical Measures

The SCD group had markedly lower blood pressures than HbAA controls: median SBP was 112 mmHg (IQR 17) versus 132 mmHg (IQR 16), *p* < 0.001; median diastolic blood pressure (DBP) was 62 mmHg (IQR 11) versus 82 mmHg (IQR 15), *p* < 0.001. Consistently, mean arterial pressure was lower in SCD (79.8 ± 8.5 mmHg) than in HbAA (97.4 ± 7.2 mmHg; *p* < 0.001). Compared with HbAA controls, participants with SCD had significantly lower BMI (21.4 ± 3.6 vs. 26.6 ± 6.6 kg/m^2^; *p* < 0.001), attributable to lower body weight. Height did not differ between groups (*p*  =  0.173) (Table 1).

### 3.3 Laboratory Measures

The mean ± SD total white blood cell (WBC) count was higher in SCD participants than in the HbAA comparison group: 10.3 ± 3.1 × 10^9^/L versus 5.1 ± 1.1 × 10^9^/L (*p* < 0.001). Mean haemoglobin was lower in SCD: 8.4 ± 1.7 g/dL versus 14.3 ± 1.4 g/dL (*p* < 0.001). Mean corpuscular volume (MCV) was comparable between groups: 80.5 ± 8.8 fL versus 82.3 ± 5.4 fL (*p* = 0.078). The absolute reticulocyte count was higher in SCD: 253.9 ± 91.1 × 10^9^/L versus 62.4 ± 27.2 × 10^9^/L (*p* < 0.001); platelet counts were also higher: 391.5 ± 154.2 × 10^9^/L versus 221.1 ± 71.7 × 10^9^/L (*p* < 0.001). Markers of haemolysis were elevated in SCD: serum LDH 882.0 ± 315.2 U/L versus 413.2 ± 103.0 U/L (*p* < 0.001); total bilirubin 53.8 ± 34.6 µmol/L versus 18.0 ± 8.1 µmol/L (*p* < 0.001). Except for MCV, all between‐group differences were statistically significant (Table 2).

**TABLE 2:** Laboratory parameters for SCD participants and HbAA comparison group.

Lab parameters	SCD (*n* = 75), mean (SD)	HbAA (*n* = 75), mean (SD)	*p*‐value
WBC (10^9^/L)	10.3 (3.1)	5.1 (1.1)	**< 0.001**
HGB (g/dL)	8.4 (1.7)	14.3 (1.4)	**< 0.001**
MCV (fL)	80.5 (8.8)	82.3 (5.4)	0.078
PLT (10^9^/L)	391.5 (154.2)	221.1 (71.7)	**< 0.001**
NEUT (10^9^/L)	5.1 (2.3)	2.3 (0.8)	**< 0.001**
Ret # (10^9^/L)	253.9 (91.1)	62.4 (27.2)	**< 0.001**
Total BILIRUBIN (µmol/L)	53.8 (34.6)	18.0 (8.1)	**< 0.001**
LDH (U/L)	882 (315.2)	413.2 (103.0)	**< 0.001**
sVCAM‐1 (ng/mL)	878.8 (220.3)	507.9 (221.2)	**< 0.001**
S‐Cr (µmol/L)	66.57 (21.5)	107.4 (21.4)	**< 0.001**
eGFR (CKD‐EPI 2021)	112.5 (23.8)	80.7 (15.67)	**< 0.001**

	HbSS (*n* = 65), mean (SD)	HbSC (*n* = 10), mean (SD)	
sVCAM‐1 (ng/mL)	890.6 (208.4)	802.4 (287.6)	0.241

*Note*: Data are mean (SD) unless otherwise indicated. Between‐group comparisons used a *t*‐test or a Mann–Whitney *U* test, as appropriate (two‐sided). Bold *p*‐values indicate *p* < 0.05.

Abbreviations: eGFR, estimated glomerular filtration rate; HGB, haemoglobin; LDH, lactate dehydrogenase; MCV, mean corpuscular volume; NEUT, neutrophil count; PLT, platelet count; Ret#, absolute reticulocyte count; S‐Cr, serum creatinine; sVCAM‐1, soluble vascular cell adhesion molecule‐1; WBC, white blood cell count.

### 3.4 Complications of Sickle Cell Disease

Complications were common among participants with SCD. The most frequent were any prior hospitalization (93.3%; 70/75), vaso‐occlusive crisis requiring an emergency room visit in the past 12 months (53.3%; 40/75), and sepsis or severe infection (48.3%; 28/58). Acute chest syndrome was reported in 26.0% (19/73) of participants, while priapism occurred in 38.5% (10/26) of male participants. Other complications, including chronic leg ulcers, avascular necrosis, transfusion‐requiring anaemia, gallstones, retinopathy and nephropathy, were less frequent. No cases of stroke, cardiomyopathy, pulmonary hypertension, hypertension or diabetes mellitus were recorded. There were no statistically significant differences in complication rates between HbSS and HbSC participants (Table S4).

### 3.5 Concentration of Serum sVCAM‐1

Compared with HbAA controls, sVCAM‐1 was markedly elevated in SCD in the steady state (878.8 ± 220.3 vs. 507.9 ± 221.2 ng/mL; *p* < 0.001). Within SCD, sVCAM‐1 did not differ between HbSS and HbSC subgroups (890.6 ± 208.4 vs. 802.4 ± 287.6 ng/mL; *p*  =  0.241) in the steady state (Table 2).

### 3.6 Association Between Serum sVCAM‐1 and Absolute Reticulocyte Count

sVCAM‐1 was positively correlated with the absolute reticulocyte count in the combined SCD cohort (*r* =  0.24, *p*  =  0.034). Genotype‐stratified analyses showed a positive trend in participants with HbSS (*r*  =  0.22, *p*  =  0.075), whereas the association in HbSC (*n*  =  10) was weak (*r*  =  0.13, *p*  =  0.715). No association was observed among HbAA controls (*r * =  −0.10, *p*  =  0.376). (Table 3 and Figure 1)

**TABLE 3:** Pearson correlation between sVCAM‐1 and demographic, clinical, and laboratory parameters.

	SCD (*n* = 75)		HbSS (*n* = 65)		HbSC (*n* = 10)		HbAA (*n* = 75)	
Parameter	*r*	*p*‐value	*r*	*p*‐value	*r*	*p*‐value	*r*	*p*‐value
Age	−0.07	0.525	0.027	0.831	−0.573	0.084	0.08	0.465
BMI (kg/m^2^)	−0.20	0.081	−0.056	0.660	−0.772	**0.009**	−0.04	0.715
SBP (mmHg)	0.09	0.463	0.264	**0.033**	−0.599	0.068	−0.03	0.792
DBP (mmHg)	−0.07	0.523	0.070	0.578	−0.532	0.114	0.15	0.195
WBC (10^9^/L)	0.03	0.789	−0.021	0.865	0.033	0.927	−0.11	0.357
HGB (g/dL)	−0.01	0.942	0.068	0.592	0.370	0.293	−0.06	0.583
MCV (fL)	0.16	0.168	0.100	0.428	0.331	0.350	−0.08	0.407
PLT (10^9^/L)	0.11	0.338	0.097	0.444	−0.055	0.879	0.03	0.813
NEUT (10^9^/L)	0.01	0.952	−0.007	0.956	−0.056	0.879	−0.05	0.647
Ret# (10^9^/L)	0.24	**0.034**	0.222	0.075	0.133	0.715	−0.10	0.376
LDH (U/L)	0.05	0.698	−0.014	0.911	−0.089	0.806	0.04	0.734
Tot Bil(µmol/L)	−0.04	0.724	−0.138	0.270	0.479	0.161	0.09	0.441
S‐Cr (µmol/L)	0.01	0.947	0.126	0.317	−0.484	0.156	−0.12	0.295
eGFR (CKD‐EPI 2021)	0.02	0.839	−0.105	0.404	0.510	0.133	0.14	0.237

*Note*: Values are Pearson correlation coefficients (*r*) with two‐sided *p*‐values. Bold *p*‐values indicate *p* < 0.05.

Abbreviations: BMI, body mass index; DBP, diastolic blood pressure; eGFR, estimated glomerular filtration rate; HGB, haemoglobin; LDH, lactate dehydrogenase; MCV, mean corpuscular volume; NEUT, neutrophil count; PLT, platelet count; Ret#, absolute reticulocyte count; S‐Cr, serum creatinine; sVCAM‐1, soluble vascular cell adhesion molecule‐1; SBP, systolic blood pressure; Tot Bil, total bilirubin; WBC, white blood cell count.

**FIGURE 1:**
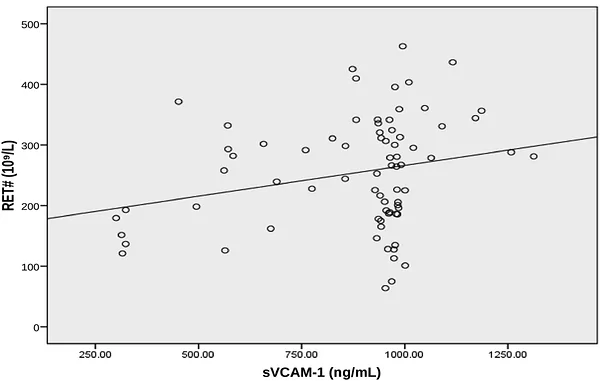
Correlation between sVCAM‐1 and absolute reticulocyte count in SCD. Scatter plot showing the relationship between serum soluble vascular cell adhesion molecule‐1 (sVCAM‐1; ng/mL) and absolute reticulocyte count (Ret#; ×10^9^/L) among adults with sickle cell disease (SCD) in steady state (*n* = 75). Each point represents one participant, and the solid line represents the fitted linear regression trend. Pearson's correlation showed a positive association between sVCAM‐1 and absolute reticulocyte count (*r* = 0.24, *p* = 0.034).

### 3.7 Association Between Serum sVCAM‐1 and Blood Pressure

Serum sVCAM‐1 showed a small positive correlation with SBP in the HbSS subgroup (*r* = 0.264, *p* = 0.033), whereas no associations with diastolic blood pressure were observed (*r* = 0.070, *p* = 0.578). In the pooled SCD cohort and in HbAA controls, sVCAM‐1 did not correlate with SBP or DBP. In the HbSC subgroup (*n* = 10), correlations were inverse and of moderate magnitude (SBP *r* = −0.599, *p* = 0.068; DBP *r* = −0.532, *p* = 0.114) but did not reach statistical significance, likely reflecting limited power. (Table 3 and Figure 2)

**FIGURE 2:**
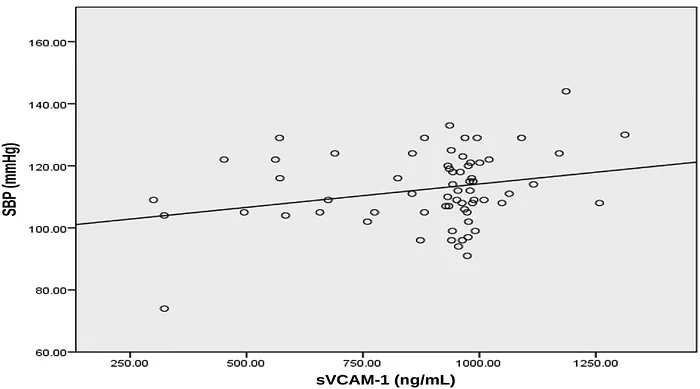
Correlation between sVCAM‐1 and SBP in HbSS. Scatter plot showing the relationship between serum soluble vascular cell adhesion molecule‐1 (sVCAM‐1; ng/mL) and systolic blood pressure (SBP; mmHg) among adults with HbSS sickle cell disease in steady state (*n* = 65). Each point represents one participant, and the solid line represents the fitted linear regression trend. Pearson's correlation demonstrated a small positive association between sVCAM‐1 and SBP (*r* = 0.264, *p* = 0.033).

### 3.8 Associations Between sVCAM‐1 and Other Demographic, Clinical and Laboratory Measures

In the combined SCD cohort, BMI showed a weak inverse trend with sVCAM‐1 (*r*  =  −0.20, *p*  =  0.081) that did not reach conventional statistical significance. Genotype‐stratified Pearson analyses showed a strong inverse correlation between BMI and sVCAM‐1 in the small HbSC subgroup (*r*  =  −0.772, *p*  =  0.009; n  =  10), whereas no association was observed in HbSS (*r*  =  −0.056, *p*  =  0.660) or HbAA (*r*  =  −0.04, *p*  =  0.715) participants. However, the HbSC association was attenuated in the Spearman sensitivity analysis (ρ = −0.37, p = 0.288) and should be considered exploratory (Table S1). Serum VCAM‐1 did not show statistically significant correlations with age, diastolic BP, WBC, HGB, MCV, platelets, neutrophils, LDH or total bilirubin in the combined SCD cohort (all *p* ≥ 0.168), with similar results in HbSS and HbAA (all *p* ≥ 0.195) (Table 3). These associations remained broadly consistent after Box–Cox transformation of sVCAM‐1 (Table S3). No complication showed a statistically significant association with sVCAM‐1 in the combined SCD cohort (Table S5). The frequency of vaso‐occlusive pain was not significantly associated with sVCAM‐1 (Table S10), and sVCAM‐1 did not differ significantly across vaso‐occlusive pain frequency categories (Table S11).

### 3.9 Association between Systolic Blood Pressure and Markers of Haemolysis

In the SCD participants, SBP correlated positively with reticulocyte count (pooled SCD: *r* = 0.255, *p* = 0.027), driven by a moderate association in the HbSS subgroup (*r* = 0.348, *p* = 0.005). SBP demonstrated no significant relationships with LDH or total bilirubin in the pooled cohort, with only a non‐significant positive trend for LDH in HbSS (*r* = 0.228, *p* = 0.067). No significant correlations were observed in HbSC or in HbAA controls (Table 4). The corresponding Spearman's sensitivity analysis yielded consistent findings (Table S2).

**TABLE 4:** Pearson correlation between systolic blood pressure and markers of haemolysis.

	SCD		HbSS		HbSC		HbAA	
Parameter	*r*	*p*‐value	*r*	*p*‐value	*r*	*p*‐value	*r*	*p*‐value
Ret# (10^9^/L)	0.255	**0.027**	0.348	**0.005**	0.307	0.389	0.130	0.297
LDH (U/L)	0.151	0.195	0.228	0.067	0.141	0.697	0.123	0.293
Tot Bil (µmol/L)	0.036	0.756	0.087	0.491	−0.241	0.503	0.013	0.913

*Note*: Values are Pearson correlation coefficients (*r*) with two‐sided *p*‐values. Bold *p*‐values indicate *p* < 0.05.

Abbreviations: LDH, lactate dehydrogenase; Ret#, absolute reticulocyte count; Tot Bil, total bilirubin.

### 3.10 Multivariable Analysis of Factors Associated With sVCAM‐1

Given the modest sample size, reduced multivariable linear regression models were used to evaluate factors independently associated with sVCAM‐1 while limiting model overfitting. The primary model included sex, systolic blood pressure, total bilirubin, reticulocyte count and BMI. In the full SCD cohort, the model was statistically significant overall and explained a modest proportion of variance in sVCAM‐1 levels (*R* ^2^ = 0.147, adjusted *R* ^2^ = 0.085, *p* = 0.047). Reticulocyte count was positively associated with sVCAM‐1 (B = 73.390 ng/mL per 100x10^9^/L, *β* = 0.303, *p* = 0.014), while BMI was inversely associated (B = −15.256, *β* = −0.250, *p* = 0.038; Table 5 and Figure 3). In the HbSS subgroup, the model showed borderline overall significance (*R* ^2^ = 0.164, adjusted *R* ^2^ = 0.093, *p* = 0.055), with total bilirubin inversely associated with sVCAM‐1 (B = −1.620, *β* = −0.276, *p* = 0.041; Table 6 and Figure S1) and reticulocyte count showing a non‐significant positive trend (B = 58.840 ng/mL per 100x10^9^/L, *β* = 0.236, *p* = 0.081). VIF values were low, indicating no substantial multicollinearity (Tables 5 and 6). Alternative models that replaced sex with age or excluded both age and sex yielded similar estimates of reticulocyte count and BMI in the combined SCD cohort (Tables S6–S9). In these models, systolic blood pressure showed a non‐significant positive trend in the HbSS subgroup.

**TABLE 5:** Multivariable linear regression of factors associated with sVCAM‐1 in participants with SCD (*N* = 75).

	Unstandardized coefficients		Standardized coeff.	*t*	Sig.	95.0% CI for B		
Model	B	Std. error	Beta	*t*	Sig.	Lower	Upper	Collinearity VIF
(Constant)	961.712	253.471		3.794	0.000	456.052	1467.373	
SEX (Male = 1)	44.113	55.637	0.096	0.793	0.431	−66.881	155.106	1.18
SBP (mmHg)	0.962	2.175	0.053	0.442	0.660	−3.378	5.301	1.17
T. Bil (µmol/L)	−1.238	0.779	−0.194	−1.588	0.117	−2.793	0.317	1.21
Ret# (100x10^9^/L)	73.390	29.197	0.303	2.514	**0.014**	15.143	131.637	1.18
BMI (kg/m^2^)	−15.256	7.218	−0.250	−2.114	**0.038**	−29.654	−.857	1.13

*Note: R* ^2^ = 0.147, Adjusted *R* ^2^ = 0.085, F(5,69) = 2.380, *p* = 0.047, AIC = 1021.2, BIC = 1035.1. Multivariable linear regression with serum sVCAM‐1 (ng/mL) as the dependent variable. The model included sex, systolic blood pressure, total bilirubin, absolute reticulocyte count, and BMI. All *p*‐values are two‐sided; bold indicates *p* < 0.05. Reticulocyte count was modelled per 100x10^9^/L.

Abbreviations: B, unstandardized regression coefficient; CI, confidence interval; SE, standard error; VIF, variance inflation factor; β, standardized regression coefficient.

**FIGURE 3:**
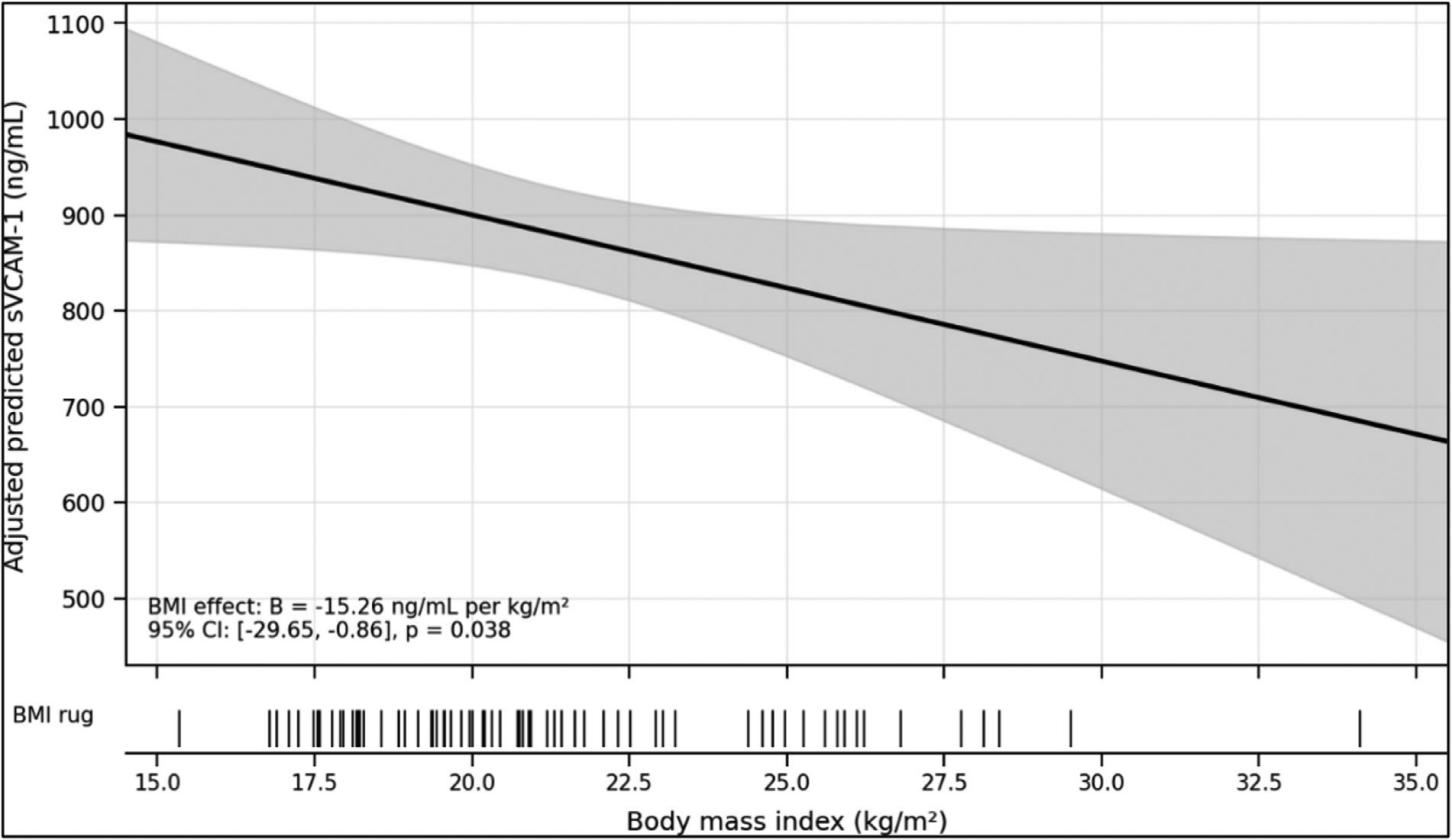
Adjusted marginal effect of BMI on sVCAM‐1 in SCD. The plot shows the adjusted predicted sVCAM‐1 levels (ng/ml) across BMI (kg/m^2^) in adults with SCD in steady state (*n* = 75). Predictions are derived from a multivariable linear regression model that adjusts for sex, absolute reticulocyte count, systolic blood pressure, and total bilirubin. The solid black line represents the estimated BMI effect, and the shaded grey band represents the 95% confidence interval. Rug marks along the *x*‐axis indicate the distribution of observed BMI. BMI remained significantly and inversely associated with sVCAM‐1 levels (B = −15.26 ng/mL per kg/m^2^, 95% CI: −29.65 to −0.86, *p* = 0.038).

**TABLE 6:** Multivariable linear regression of factors associated with sVCAM‐1 in participants with HbSS (*N* = 65).

	Unstandardized coefficients		Standardized coeff.	*t*	Sig.	95.0% CI for B		
Model	B	Std. error	Beta	*t*	Sig.	Lower	Upper	Collinearity VIF
(Constant)	555.127	272.770		2.035	0.046	9.314	1100.939	
SEX (Male = 1)	67.570	59.854	0.153	1.129	0.264	−52.197	187.336	1.29
SBP (mmHg)	3.373	2.273	0.192	1.484	0.143	−1.175	7.922	1.19
T. Bil (µmol/L)	−1.620	0.776	−0.276	−2.087	**0.041**	−3.173	−0.067	1.23
Ret# (100x10^9^/L)	58.840	33.106	0.236	1.777	0.081	−7.406	125.085	1.24
BMI (kg/m^2^)	−6.301	7.514	−0.107	−0.839	0.405	−21.336	8.734	1.14

*Note: R* ^2^ = 0.164, Adjusted *R* ^2^ = 0.093, F(5,59) = 2.316, *p* = 0.055, AIC = 877.9, BIC = 891.0. Multiple linear regression restricted to HbSS with sVCAM‐1 as the dependent variable. Two‐sided *p*‐values; bold indicates *p* < 0.05. Analysis restricted to participants with HbSS. Reticulocyte count was modelled per 100x10^9^/L.

Abbreviations: 95% CI, 95% confidence interval; B, unstandardized coefficient; Beta, standardized coefficient; BMI, body mass index; Ret#, absolute reticulocyte count; SE, standard error; sVCAM‐1, soluble vascular cell adhesion molecule‐1; SBP, systolic blood pressure; T. Bil, total bilirubin.

## 4 Discussion

This study evaluated clinical and haematologic correlates of serum sVCAM‐1, a surrogate marker of endothelial activation, in adults with steady‐state SCD. Rather than establishing that sVCAM‐1 is elevated in SCD, as previously reported in both international and Ghanaian cohorts [11, 17], our study focused on identifying clinical and haematologic correlates of sVCAM‐1 in a steady‐state adult SCD population.

Reticulocyte count emerged as the most consistent haematologic correlate of sVCAM‐1, showing strong positive associations with systolic blood pressure in both the combined SCD cohort (HbSS + HbSC) and the HbSS‐only subgroup. In the combined SCD cohort, it correlated positively with sVCAM‐1 and was independently associated with higher sVCAM‐1 in multivariable models, with a similar borderline signal in HbSS subgroup. These findings align with the well‐established roles of haemolysis and reticulocytosis in promoting vascular dysfunction by enhancing red blood cell adhesion and endothelial stress. These associations with sVCAM‐1 and systolic blood pressure support further evaluation of reticulocyte count as a readily available correlate of endothelial activation.

An inverse association between bilirubin and sVCAM‐1 was observed in the primary HbSS model, although it was attenuated and no longer statistically significant in sensitivity models. This finding should therefore be regarded as exploratory. Bilirubin has recognized antioxidant and anti‐inflammatory properties [30, 31, 32], mechanisms expected to reduce endothelial activation. Conversely, LDH, another haemolysis marker, was not significantly associated with sVCAM‐1, highlighting potential differences in how specific haemolytic pathways influence endothelial stress. The routine laboratory determination of serum LDH includes all five of its isoenzymes, which are non‐uniformly distributed across body tissues [33]. Although used as a marker of haemolysis, LDH elevation reflects non‐specific tissue damage, making it a non‐specific marker of haemolysis [34], which may explain its weaker relationship with sVCAM‐1 i.

In multivariable analysis, lower BMI was independently associated with higher sVCAM‐1 in the combined SCD cohort, and this association remained broadly consistent across alternative regression models. This finding suggests a possible relationship between body habitus and endothelial activation in SCD. Lower BMI may reflect differences in nutritional status, body composition, metabolic demand or overall disease burden [24, 35, 36]; however, nutritional intake, body composition and metabolic parameters were not directly measured in this study; therefore, the mechanisms underlying the observed association cannot be determined. Undernutrition and impaired growth are well‐documented complications of SCD, particularly in African cohorts, and have been linked to increased disease severity and poorer outcomes [3, 37, 38]. The particularly strong inverse Pearson's correlation between BMI and sVCAM‐1 observed in the HbSC subgroup should be interpreted with caution, given the small sample size (*n* = 10) and the attenuation of the association in the Spearman's sensitivity analysis. Larger studies with direct assessment of nutritional and metabolic status are needed to determine whether BMI is independently related to endothelial activation in SCD.

Systolic blood pressure was positively associated with sVCAM‐1 in univariate analyses among HbSS participants, although this relationship weakened after multivariable adjustment. These findings suggest a potential relationship between vascular haemodynamics and endothelial activation in HbSS; however, the observed effects were modest and require validation in larger studies. Endothelial dysfunction can precede and contribute to the development of hypertension by increasing peripheral resistance and promoting small‐artery remodelling [39, 40]. Among the phenotypic characteristics of endothelial dysfunction is the up‐regulation of sVCAM‐1 [41]. Notably, within the HbSS subgroup, systolic blood pressure was significantly and positively associated with the absolute reticulocyte count, a marker for haemolytic drive that enhances adhesive interactions.

Together, these findings suggest that endothelial activation in SCD reflects a multifactorial process shaped by haemolysis, nutritional status, antioxidant capacity and vascular regulation, with potential genotype‐specific differences.

### 4.1 Strengths and Limitations

A strength of this study is its evaluation of clinical and haematological correlates of sVCAM‐1 in an adult sub‐Saharan African cohort, where the burden of SCD is high, but data on endothelial biology remain limited. By including both the overall cohort and the HbSS subgroup, the analysis highlights consistent patterns while accounting for genotype‐specific differences.

Several limitations should be acknowledged. First, the cross‐sectional design precludes causal inference and assessment of temporal relationships. Second, only sVCAM‐1 was assessed, limiting evaluation of the broader endothelial activation and injury pathways involved in SCD. Third, the small HbSC subgroup (*n* = 10) reduced statistical power, rendering genotype‐specific findings exploratory. Fourth, the marked sex imbalance between the SCD and predominantly male HbAA blood‐donor groups may have influenced between‐group comparisons. Finally, the unavailability of systematically collected data on HbS, HbF, inflammatory biomarkers, lipid profiles, smoking status and other clinical and biological factors may have contributed to residual confounding.

### 4.2 Clinical Implications

The association between a higher reticulocyte count and higher sVCAM‐1 is potentially relevant because the reticulocyte count is routinely available in the clinical assessment of patients with SCD and may serve as a practical correlate of haemolytic activity and endothelial activation. However, the present cross‐sectional findings do not establish its utility for vascular risk stratification. Prospective studies are needed to determine whether reticulocyte count is associated with longitudinal changes in endothelial activation or predicts vascular complications and other clinically relevant outcomes.

The observed associations of sVCAM‐1 with BMI, bilirubin and systolic blood pressure provide additional hypothesis‐generating signals regarding the potential contributions of nutritional or metabolic status, antioxidant pathways and vascular haemodynamics to endothelial activation in SCD. These associations require validation in larger, adequately powered cohorts, particularly for genotype‐specific analyses. Future studies incorporating a broader range of endothelial and inflammatory biomarkers, quantitative haemoglobin fractions and longitudinal clinical outcomes may help clarify the biological and clinical significance of these relationships.

## 5 Conclusion

Reticulocytosis was consistently associated with higher sVCAM‐1 levels in adults with steady‐state SCD, supporting a relationship between erythropoietic/haemolytic activity and endothelial activation. Associations of sVCAM‐1 with BMI, bilirubin and systolic blood pressure were observed but should be interpreted cautiously, particularly in genotype‐specific analyses. Larger prospective studies incorporating a broader range of endothelial, inflammatory and haematologic biomarkers are needed to validate these associations and determine their clinical significance. These findings support further prospective evaluation of the relationships between haemolytic activity, nutritional status, vascular physiology and endothelial activation in SCD.

## Author Contributions

E.O. and W.K.G. designed the study. W.K.G. and W.D‐A. collected the data. E.O., W.K.G. and E.V.A. verified genotype and clinical data from patient records. G.O.A., E.K‐B. and M.F.O. contributed essential reagents and tools. W.K.G., E.V.A., J.B.A‐N., E.O. and G.O.A. performed the analyses. W.K.G., E.V.A., J.B.A‐N., E.O. and G.O.A. interpreted the results. All authors participated in writing the manuscript and reviewed and approved the final manuscript before submission.

## Funding

The study was supported by the Danida Fellowship Centre through the Building Stronger Universities Phase 3 Project at the University of Ghana.

## Ethics Statement

This study was approved by the Scientific and Technical Committee and the Institutional Review Board of the Korle‐Bu Teaching Hospital (KBTH‐STC/IRB/000217/2021). In addition, institutional approvals were obtained from the Ghana Institute of Clinical Genetics (GICG) and the Southern Area Blood Centre of the National Blood Service of Ghana.

## Consent

Participants (or their legal representatives) provided written informed consent for inclusion in this study and for publication of aggregate, de‐identified results. No individual‐level identifying information or images are included in the article.

## Conflicts of Interest

The authors declare no conflicts of interest.

## Supporting information




**Supporting Information**: jha270413‐sup‐0001‐SuppMat.docx

## Data Availability

The data that support the findings of this study are available on request from the corresponding author. The data are not publicly available due to privacy or ethical restrictions.
