# Supplementary material for: Clinical and Haematologic Correlates of Soluble VCAM‐1 in Adults With Steady‐State Sickle Cell Disease
Source: EJHaem. 2026 Oct 22;7(5):e70413. doi: 10.1002/jha2.70413 (PMC13690017; doi:10.1002/jha2.70413)
Supplement: Supplementary file 1 [file JHA2-7-e70413-s001.docx]

**Clinical and Haematologic Correlates of Soluble VCAM-1 in Adults with Steady-State Sickle Cell Disease**

**SUPPLEMENTARY TABLES**

**Table S1: Spearman’s correlation (ρ) between sVCAM-1 and Demographic, clinical, and laboratory parameters for the SCD participants and HbAA comparison group**

| Parameter | SCD (n=75) | | HbSS (n=65) | | HbSC (n=10) | | HbAA (n=75) | |
| --- | --- | --- | --- | --- | --- | --- | --- | --- |
|  | **ρ** | **p-value** | **ρ** | **p-value** | **ρ** | **p-value** | **ρ** | **p-value** |
| Age | -0.10 | 0.382 | -0.01 | 0.934 | -0.63 | 0.051 | 0.05 | 0.697 |
| BMI (kg/m²) | -0.17 | 0.139 | -0.11 | 0.373 | -0.37 | 0.288 | -0.07 | 0.521 |
| SBP (mmHg) | 0.10 | 0.382 | 0.19 | 0.118 | -0.32 | 0.359 | 0.04 | 0.759 |
| DBP (mmHg) | 0.004 | 0.970 | 0.06 | 0.618 | -0.23 | 0.525 | 0.18 | 0.123 |
| WBC (10^9^/L) | 0.07 | 0.551 | 0.04 | 0.770 | -0.36 | 0.305 | -0.09 | 0.425 |
| HGB(g/dL) | 0.03 | 0.777 | 0.05 | 0.698 | 0.24 | 0.503 | -0.07 | 0.548 |
| MCV (fL) | 0.07 | 0.528 | 0.07 | 0.591 | 0.29 | 0.398 | -0.16 | 0.159 |
| PLT (10^9^/L) | 0.06 | 0.602 | 0.08 | 0.531 | 0.13 | 0.722 | 0.04 | 0.736 |
| NEUT (10^9^/L) | 0.08 | 0.493 | 0.11 | 0.393 | -0.42 | 0.225 | -0.007 | 0.954 |
| RET# (10⁹/L) | 0.22 | 0.063 | 0.22 | 0.085 | 0.24 | 0.505 | -0.006 | 0.958 |
| LDH (U/L) | 0.04 | 0.708 | 0.04 | 0.730 | 0.08 | 0.826 | -0.02 | 0.870 |
| Tot Bil(µmol/L) | 0.02 | 0.890 | -0.11 | 0.403 | 0.69 | 0.028 | 0.12 | 0.289 |
| S-Cr (µmol/L) | 0.14 | 0.23 | 0.26 | **0.040** | -0.46 | 0.19 | -0.08 | 0.507 |
| eGFR | -0.01 | 0.917 | -0.13 | 0.287 | 0.58 | 0.082 | 0.12 | 0.325 |

**Sensitivity analysis:** Spearman rank correlations were performed to assess the robustness of the primary Pearson correlation findings to departures from linearity and the potential influence of individual observations. In the combined SCD and HbSS cohorts, the direction and magnitude of most associations were broadly similar to those observed in the primary Pearson analyses. In the small HbSC subgroup (n=10), however, the strong inverse Pearson correlation between BMI and sVCAM-1 (r=−0.772, p=0.009) was attenuated in the Spearman analysis (ρ=−0.37, p=0.288). This difference indicates that the HbSC BMI–sVCAM-1 association is sensitive to the analytic method and should therefore be considered exploratory

**Table S2: Spearman’s correlation between Systolic Blood Pressure and Markers of Haemolysis**

| Parameter | SCD | | HbSS | | HbSC | | HbAA | |
| --- | --- | --- | --- | --- | --- | --- | --- | --- |
|  | **ρ** | **p-value** | **ρ** | **p-value** | **ρ** | **p-value** | **ρ** | **p-value** |
| RET# (10⁹/L) | 0.24 | **0.036** | 0.34 | **0.006** | 0.307 | 0.389 | 0.16 | 0.158 |
| LDH (U/L) | 0.14 | 0.246 | 0.22 | 0.076 | 0.141 | 0.697 | 0.20 | 0.080 |
| Tot Bil(µmol/L) | -0.009 | 0.934 | 0.07 | 0.551 | -0.241 | 0.503 | 0.03 | 0.812 |

Spearman’s rank correlation coefficients (ρ) and corresponding *p*-values are shown for the overall SCD group and the HbSS, HbSC, and HbAA groups. Systolic blood pressure was positively correlated with absolute reticulocyte count in the overall SCD group and the HbSS subgroup (*p* < 0.05); the remaining correlations were not statistically significant.

**Table S3: Pearson's correlation for box-cox transformed sVCAM-1 and Demographic, clinical, and laboratory parameters for the SCD participants and HbAA comparison group**

| Parameter | SCD (n=75) | | | HbSS (n=65) | | HbSC (n=10) | | HbAA (n=75) | |
| --- | --- | --- | --- | --- | --- | --- | --- | --- | --- |
|  | **r** | **p-value** | | **r** | **p-value** | **r** | **p-value** | **r** | **p-value** |
| Age | -0.08 | 0.465 | | -0.005 | 0.967 | -0.58 | 0.07 | 0.10 | 0.382 |
| BMI (kg/m²) | -0.19 | 0.098 | | -0.07 | 0.551 | -0.76 | **0.011** | 0.002 | 0.985 |
| SBP (mmHg) | 0.12 | 0.292 | | 0.27 | **0.03** | -0.62 | 0.057 | 0.03 | 0.777 |
| DBP (mmHg) | -0.06 | 0.573 | | 0.05 | 0.663 | -0.56 | 0.093 | 0.10 | 0.383 |
| WBC (10^9^/L) | 0.01 | 0.921 | | -0.04 | 0.771 | 0.04 | 0.907 | -0.10 | 0.376 |
| HGB(g/dL) | 0.005 | 0.963 | | 0.07 | 0.566 | 0.38 | 0.278 | -0.04 | 0.735 |
| MCV (fL) | 0.12 | 0.291 | | 0.07 | 0.573 | 0.28 | 0.432 | -0.07 | 0.525 |
| PLT (10^9^/L) | 0.07 | 0.532 | | 0.06 | 0.644 | -0.11 | 0.772 | 0.00 | 0.999 |
| NEUT (10^9^/L) | -0.0007 | | 0.995 | -0.02 | 0.901 | -0.04 | 0.909 | -0.04 | 0.752 |
| RET# (10⁹/L) | 0.24 | **0.040** | | 0.22 | 0.082 | 0.15 | 0.683 | -0.09 | 0.423 |
| LDH (U/L) | 0.05 | 0.655 | | 0.003 | 0.984 | -0.04 | 0.905 | 0.04 | 0.746 |
| Tot Bil(µmol/L) | -0.06 | 0.607 | | -0.14 | 0.258 | 0.51 | 0.134 | 0.07 | 0.513 |
| S-Cr (µmol/L) | 0.03 | 0.834 | | 0.13 | 0.310 | -0.54 | 0.105 | -0.13 | 0.260 |
| eGFR | 0.03 | 0.829 | | -0.09 | 0.468 | 0.58 | 0.077 | 0.13 | 0.269 |

Because the distribution of sVCAM-1 was asymmetric, a Box–Cox transformation was applied and Pearson correlations were repeated using the transformed outcome. Results were generally consistent with the primary Pearson analyses. In the combined SCD cohort, reticulocyte count remained positively correlated with sVCAM-1 (r=0.24, p=0.040), while in HbSS, systolic blood pressure remained positively correlated with sVCAM-1 (r=0.27, p=0.030). In HbSC (n=10), the strong inverse Pearson association between BMI and sVCAM-1 persisted after Box–Cox transformation (r=−0.76, p=0.011); however, this association was substantially attenuated in the Spearman rank analysis (ρ=−0.37, p=0.288; Table S1). Given the small HbSC sample and sensitivity of this association to the correlation method, the HbSC finding should be considered exploratory.

**Table S4: Complications of Sickle Cell Disease in the Study Participants**

| Complication | SCD (N=75) | HbSS (N=65) | HbSC (N=10) | Fisher p (SS vs SC) |
| --- | --- | --- | --- | --- |
| VOC requiring ER visit, past 12 months | 40/75 (53.3%) | 35/65 (53.8%) | 5/10 (50.0%) | 1.000 |
| History of Acute Chest Syndrome | 19/73 (26.0%) | 18/63 (28.6%) | 1/10 (10.0%) | 0.437 |
| Hx of anaemia requiring transfusion | 10/75 (13.3%) | 10/65 (15.4%) | 0/10 (0.0%) | 0.340 |
| Avascular necrosis (femoral/humeral) | 12/75 (16.0%) | 11/65 (16.9%) | 1/10 (10.0%) | 1.000 |
| Chronic leg ulcers | 14/75 (18.7%) | 13/65 (20.0%) | 1/10 (10.0%) | 0.677 |
| Nephropathy (persistent proteinuria) | 4/75 (5.3%) | 4/65 (6.2%) | 0/10 (0.0%) | 1.000 |
| Stroke | 0/75 (0.0%) | 0/65 (0.0%) | 0/10 (0.0%) | NE |
| Sepsis / severe infection | 28/58 (48.3%) | 23/50 (46.0%) | 5/8 (62.5%) | 0.464 |
| Gallstones | 9/75 (12.0%) | 7/65 (10.8%) | 2/10 (20.0%) | 0.344 |
| Priapism (male participants only, n=26) | 10/26 (38.5%) | 9/21 (42.9%) | 1/5 (20.0%) | 0.617 |
| Retinopathy | 6/75 (8.0%) | 5/65 (7.7%) | 1/10 (10.0%) | 1.000 |
| Cardiomyopathy | 0/75 (0.0%) | 0/65 (0.0%) | 0/10 (0.0%) | NE |
| Any hospitalization (ever) | 70/75 (93.3%) | 62/65 (95.4%) | 8/10 (80.0%) | 0.129 |
| Pulmonary hypertension | 0/75 (0.0%) | 0/65 (0.0%) | 0/10 (0.0%) | NE |
| Other Medical Conditions |  | | | |
| Hypertension | 0/75 (0.0%) | 0/65 (0.0%) | 0/10 (0.0%) | NE |
| Diabetes mellitus | 0/75 (0.0%) | 0/65 (0.0%) | 0/10 (0.0%) | NE |

Data are presented as *n/N* (%). Denominators vary because “don’t know” responses were excluded on a complication-specific basis. Differences between the HbSS and HbSC groups were assessed using Fisher’s exact test. Priapism was evaluated in male participants only. NE, not estimable because no events occurred in either genotype group

**Table S5: Association between clinical complications of SCD and serum VCAM-1**

| Clinical complication | Cohort | N | Present, n | Pearson r (p) | Spearman rho (p) | Box-Cox r (p) |
| --- | --- | --- | --- | --- | --- | --- |
| VOC in the past 12 months | Combined SCD | 75 | 40 | -0.058 (0.619) | -0.193 (0.097) | -0.088 (0.454) |
|  | HbSS | 65 | 35 | -0.012 (0.924) | -0.174 (0.166) | -0.048 (0.703) |
|  | HbSC | 10 | 5 | -0.316 (0.373) | -0.282 (0.430) | -0.362 (0.304) |
| History of Acute Chest Syndrome | Combined SCD | 73 | 19 | -0.024 (0.843) | -0.109 (0.359) | -0.057 (0.634) |
|  | HbSS | 63 | 18 | -0.078 (0.546) | -0.123 (0.338) | -0.102 (0.428) |
|  | HbSC | 10 | 1 | 0.194 (0.591) | -0.058 (0.873) | 0.183 (0.613) |
| History of anaemia requiring transfusion | Combined SCD | 75 | 10 | -0.082 (0.482) | -0.204 (0.079) | -0.129 (0.271) |
|  | HbSS | 65 | 10 | -0.119 (0.345) | -0.228 (0.067) | -0.159 (0.205) |
|  | HbSC | 10 | 0 | NE | NE | NE |
| Avascular necrosis | Combined SCD | 75 | 12 | 0.033 (0.776) | -0.027 (0.819) | -0.009 (0.941) |
|  | HbSS | 65 | 11 | -0.002 (0.986) | -0.039 (0.756) | -0.040 (0.750) |
|  | HbSC | 10 | 1 | 0.194 (0.591) | -0.058 (0.873) | 0.183 (0.613) |
| Chronic leg ulcers | Combined SCD | 75 | 14 | 0.091 (0.436) | 0.102 (0.384) | 0.070 (0.553) |
|  | HbSS | 65 | 13 | 0.065 (0.608) | 0.108 (0.393) | 0.046 (0.713) |
|  | HbSC | 10 | 1 | 0.194 (0.591) | -0.058 (0.873) | 0.183 (0.613) |
| Nephropathy (Persistent proteinuria) | Combined SCD | 75 | 4 | -0.042 (0.720) | -0.071 (0.543) | -0.056 (0.633) |
|  | HbSS | 65 | 4 | -0.063 (0.621) | -0.078 (0.534) | -0.073 (0.566) |
|  | HbSC | 10 | 0 | NE | NE | NE |
| Sepsis | Combined SCD | 58 | 28 | 0.235 (0.075) | 0.148 (0.266) | 0.223 (0.093) |
|  | HbSS | 50 | 23 | 0.228 (0.112) | 0.099 (0.495) | 0.219 (0.126) |
|  | HbSC | 8 | 5 | 0.320 (0.439) | 0.620 (0.101) | 0.279 (0.503) |
| Gallstones | Combined SCD | 75 | 9 | 0.021 (0.857) | 0.074 (0.529) | 0.029 (0.805) |
|  | HbSS | 65 | 7 | -0.042 (0.741) | 0.013 (0.917) | -0.023 (0.858) |
|  | HbSC | 10 | 2 | 0.328 (0.356) | 0.348 (0.324) | 0.343 (0.331) |
| Priapism (males only) | Combined SCD | 26 | 10 | 0.161 (0.431) | 0.200 (0.327) | 0.143 (0.484) |
|  | HbSS | 21 | 9 | 0.121 (0.602) | 0.222 (0.332) | 0.114 (0.623) |
|  | HbSC | 5 | 1 | 0.220 (0.722) | -0.354 (0.559) | 0.166 (0.790) |
| Retinopathy | Combined SCD | 75 | 6 | -0.078 (0.505) | -0.168 (0.150) | -0.108 (0.359) |
|  | HbSS | 65 | 5 | -0.026 (0.838) | -0.117 (0.354) | -0.052 (0.680) |
|  | HbSC | 10 | 1 | -0.291 (0.415) | -0.290 (0.416) | -0.411 (0.237) |
| Any hospitalization | Combined SCD | 75 | 70 | -0.159 (0.173) | -0.133 (0.254) | -0.170 (0.145) |
|  | HbSS | 65 | 62 | -0.160 (0.202) | -0.160 (0.202) | -0.184 (0.143) |
|  | HbSC | 10 | 8 | -0.290 (0.416) | 0.087 (0.811) | -0.273 (0.446) |

Values are the correlation coefficient (two-sided p-value). N is the number of complete observations available for each complication and cohort; “don’t know” responses were excluded. Present, n denotes the number with the complication; for VOC, it denotes at least one episode, although the correlation used the reported episode count. Pearson correlations for binary complications are point-biserial correlations. Box-Cox r is the Pearson correlation after transforming VCAM-1 with a lambda estimated separately for each complication-specific cohort. Priapism analyses include males only. NE, not estimable because the complication had no variation. None of the complications was significantly associated with serum VCAM-1 at the conventional two-sided p < 0.05 threshold.

**ADDITIONAL REGRESSION MODELS (SENSITIVITY ANALYSES)**

Sensitivity analyses were performed to assess the robustness of the primary multivariable regression findings to alternative covariate specifications. For the combined SCD and HbSS cohorts, two alternative models were examined: (1) a model including age, systolic blood pressure, total bilirubin, absolute reticulocyte count, and BMI; and (2) a more parsimonious model including systolic blood pressure, total bilirubin, absolute reticulocyte count, and BMI, without age or sex. Serum sVCAM-1 was the dependent variable in all models. Model fit and collinearity statistics, including R², adjusted R², AIC, BIC, and variance inflation factors (VIFs), are presented with each model in Tables S6–S9.

**Table S6. Sensitivity analysis of factors associated with sVCAM-1 in participants with SCD: model substituting age for sex (N=75)**

| Model | Unstandardized Coefficients | | Standardized Coeff. | t | Sig. | 95.0% CI for B | | Collinearity  VIF |
| --- | --- | --- | --- | --- | --- | --- | --- | --- |
|  | **B** | **Std. Error** | **Beta** | **t** | **Sig.** | **Lower** | **Upper** |  |
| (Constant) | 975.688 | 265.057 |  | 3.681 | .000 | 446.913 | 1504.462 |  |
| AGE (years) | -.773 | 2.925 | -.031 | -.264 | .792 | -6.607 | 5.062 | 1.12 |
| SBP (mmHg) | 1.433 | 2.150 | .079 | .666 | .507 | -2.857 | 5.723 | 1.14 |
| T. Bil (µmol/L) | -1.101 | .762 | -.173 | -1.444 | .153 | -2.621 | .420 | 1.15 |
| Ret# (100x10⁹/L) | 68.672 | 29.800 | .284 | 2.304 | **.024** | 9.223 | 128.121 | 1.22 |
| BMI (kg/m²) | -16.310 | 7.108 | -.267 | -2.294 | **.025** | -30.491 | -2.129 | 1.01 |

***R² = 0.140, Adjusted R² = 0.078, F(5,69) = 2.250, p = 0.059. AIC = 1021.8, BIC = 1035.7.***

**Table S7. Sensitivity analysis of factors associated with sVCAM-1 in participants with HbSS: model substituting age for sex (N=65)**

| Model | Unstandardized Coefficients | | Standardized Coeff. | t | Sig. | 95.0% CI for B | | Collinearity  VIF |
| --- | --- | --- | --- | --- | --- | --- | --- | --- |
|  | **B** | **Std. Error** | **Beta** | **t** | **Sig.** | **Lower** | **Upper** |  |
| (Constant) | 545.773 | 284.984 |  | 1.915 | .060 | -24.479 | 1116.024 |  |
| AGE (years) | .579 | 2.907 | .025 | .199 | .843 | -5.238 | 6.395 | 1.09 |
| SBP (mmHg) | 3.811 | 2.279 | .217 | 1.672 | .100 | -.750 | 8.371 | 1.17 |
| T. Bil (µmol/L) | -1.294 | .740 | -.220 | -1.748 | .086 | -2.774 | .187 | 1.10 |
| RET# (100x10⁹/L) | 55.970 | 34.091 | .224 | 1.642 | .106 | -12.246 | 124.185 | 1.29 |
| BMI (kg/m²) | -8.527 | 7.343 | -.144 | -1.161 | .250 | -23.221 | 6.166 | 1.07 |

***R² = 0.147, Adjusted R² = 0.074, F(5,59) = 2.027, p = 0.088. AIC = 879.3, BIC = 892.3.***

**Table S8. Sensitivity analysis of factors associated with sVCAM-1 in participants with SCD: parsimonious model excluding age and sex (N=75)**

| Model | Unstandardized Coefficients | | Standardized Coeff. | t | Sig. | 95.0% CI for B | | Collinearity  VIF |
| --- | --- | --- | --- | --- | --- | --- | --- | --- |
|  | **B** | **Std. Error** | **Beta** | **t** | **Sig.** | **Lower** | **Upper** |  |
| (Constant) | 956.024 | 252.696 |  | 3.783 | .000 | 452.037 | 1460.011 |  |
| SBP (mmHg) | 1.351 | 2.114 | .075 | .639 | .525 | -2.864 | 5.566 | 1.11 |
| T. Bil (µmol/L) | -1.069 | .748 | -.168 | -1.430 | .157 | -2.560 | .422 | 1.12 |
| Ret# (100x10⁹/L) | 70.403 | 28.877 | .291 | 2.438 | **.017** | 12.811 | 127.996 | 1.16 |
| BMI (kg/m²) | -16.401 | 7.053 | -.269 | -2.325 | **.023** | -30.467 | -2.334 | 1.09 |

***R² = 0.139, Adjusted R² = 0.090, F(4,70) = 2.833, p = 0.031. AIC = 1019.8, BIC = 1031.4.***

**Table S9. Sensitivity analysis of factors associated with sVCAM-1 in participants with HbSS: parsimonious model excluding age and sex (N=65)**

| Model | Unstandardized Coefficients | | Standardized Coeff. | t | Sig. | 95.0% CI for B | | Collinearity  VIF |
| --- | --- | --- | --- | --- | --- | --- | --- | --- |
|  | **B** | **Std. Error** | **Beta** | **t** | **Sig.** | **Lower** | **Upper** |  |
| (Constant) | 560.233 | 273.356 |  | 2.049 | .045 | 13.440 | 1107.027 |  |
| SBP (mmHg) | 3.882 | 2.233 | .221 | 1.738 | .087 | -.585 | 8.348 | 1.14 |
| T. Bil (µmol/L) | -1.312 | .728 | -.224 | -1.802 | .077 | -2.769 | .145 | 1.08 |
| RET# (100x10⁹/L) | 54.445 | 32.952 | .218 | 1.652 | .104 | -11.469 | 120.358 | 1.23 |
| BMI (kg/m²) | -8.475 | 7.279 | -.143 | -1.164 | .249 | -23.036 | 6.086 | 1.07 |

***R² = 0.146, Adjusted R² = 0.089, F(4,60) = 2.565, p = 0.047. AIC = 877.3, BIC = 888.2.***

Across the alternative model specifications, the principal findings in the combined SCD cohort were broadly consistent: higher absolute reticulocyte count remained positively associated with sVCAM-1, while higher BMI remained inversely associated with sVCAM-1 (Tables S6 and S8). In the HbSS subgroup, none of the individual covariates reached statistical significance in the alternative models, although systolic blood pressure remained positively associated and total bilirubin inversely associated with sVCAM-1 in direction (Tables S7 and S9). VIF values were low across all models, providing no evidence of substantial multicollinearity.

**FREQUENCY OF VASO-OCCLUSIVE CRISIS AND SERUM VCAM-1**

**Table S10:** **Correlation between number of VOC episodes and serum VCAM-1 in patients with SCD**

| Cohort | n | Pearson r | Pearson p | Spearman rho | Spearman p | Box-Cox Pearson r | Box-Cox p |
| --- | --- | --- | --- | --- | --- | --- | --- |
| SCD | 75 | -0.058 | 0.619 | -0.193 | 0.097 | -0.046 | 0.692 |
| HbSS | 65 | -0.012 | 0.924 | -0.174 | 0.166 | -0.006 | 0.962 |
| HbSC | 10 | -0.316 | 0.373 | -0.282 | 0.430 | -0.281 | 0.431 |

VOC frequency was analysed as the number of vaso-occlusive crisis episodes reported in the previous 12 months. The category >5 VOC episodes was coded as 6 for numerical correlation analyses. Pearson correlation assessed linear association, Spearman correlation assessed rank-based monotonic association, and a Box-Cox-transformed Pearson correlation was used for sensitivity analysis. There was no statistically significant association between the number of VOCs and serum VCAM-1 in the combined SCD cohort or genotype subgroups.

**Table S11: Serum VCAM-1 levels across reported VOC-frequency categories**

| VOC episodes in the last 12 months | n | Mean VCAM-1 | SD | Median VCAM-1 | IQR |
| --- | --- | --- | --- | --- | --- |
| 0 | 35 | 871.4 | 231.3 | 968.3 | 907.1 to 984.2 |
| 1 | 15 | 953.5 | 244.7 | 973.9 | 939.3 to 1037.0 |
| 2 | 16 | 865.5 | 126.8 | 929.9 | 831.5 to 945.3 |
| 3 | 5 | 762.3 | 321.8 | 856.2 | 564.5 to 961.3 |
| >5 | 4 | 862.8 | 201.6 | 950.2 | 842.7 to 970.3 |

***Kruskal-Wallis test across VOC-frequency categories: H = 7.266, p = 0.122.***

Serum VCAM-1 levels (ng/ml) are summarized across the reported VOC-frequency categories. Values are shown as mean, standard deviation (SD), median, and interquartile range (IQR). The Kruskal-Wallis test was used to assess whether VCAM-1 levels differed across VOC-frequency categories. No significant difference in serum VCAM-1 levels was observed across VOC-frequency groups. No participant had a VOC frequency of 4 or 5 over the last 12 months

**SUPPLEMENTARY FIGURE**

**Figure S1: Adjusted marginal effect of total bilirubin on sVCAM-1 in participants with HbSS (N = 65)**

The plot shows the adjusted predicted sVCAM-1 levels (ng/ml) across total bilirubin (µmol/L) in adults with HbSS in steady state (**n = 65**). Predictions were adjusted for sex, systolic blood pressure, absolute reticulocyte count, and body mass index. The solid line shows adjusted predicted sVCAM-1 concentrations, the shaded band indicates the 95% confidence interval, and rug marks show the observed distribution of total bilirubin. Total bilirubin was inversely associated with sVCAM-1 **(B = −1.62 ng/mL per µmol/L; 95% CI, −3.17 to −0.07; p = 0.041)**
